# AlScN Film Based Piezoelectric Micromechanical Ultrasonic Transducer for an Extended Long-Range Detection

**DOI:** 10.3390/mi13111942

**Published:** 2022-11-10

**Authors:** Haolin Yang, Meilin Ji, Xueying Xiu, Haochen Lv, Alex Gu, Songsong Zhang

**Affiliations:** 1 School of Microelectronics, Shanghai University, Shanghai 200444, China; 2 Shanghai Industrial μTechnology Research Institute, Shanghai 201800, China

**Keywords:** PMUT, AlScN, MEMS, long-range detection, ultrasound transducers, rangefinder, range finding

## Abstract

Piezoelectric micromachined ultrasonic transducers (PMUTs) have been widely applied in distance sensing. However, the sensing distance of currently reported miniaturized ultrasonic sensors (e.g., PMUTs or CMUT) is still limited up to a certain range (e.g., ≤5 m) compared to conventional bulk ultrasonic devices. This paper reports a PMUT array design using scandium-doped aluminum nitride (AlScN) as its piezoelectric layer for an extended long-range detection purpose. To minimize air attenuation, our device is resonating at 66 kHz for a high receive sensitivity of 5.7 mV/Pa. The proposed PMUT array can generate a sound pressure level (SPL) as high as 120 dB at a distance of 10 cm without beam forming. This PMUT design is catered for a pin-to-pin replacement of the current commercial bulk ultrasonic ranging sensor and works directly with the conventional range finding system (e.g., TI PGA460). In comparison with the common bulk transducer, the size of our device is 80% smaller. With the identical ranging detection setup, the proposed PMUT array improves the system SNR by more than 5 dB even at a distance as far as 6.8 m. The result of extended sensing distance validates our miniaturized PMUT array as the optimized candidate for most ultrasonic ranging applications. With the progressive development of piezoelectric MEMS, we believe that the PMUT technology could be a game changer in future long-range sensing applications.

## 1. Introduction

Range finding is a basic system-identification problem in which a sensor seeks to determine the location of other objects in the sensor’s surroundings [1]. Recently, the rangefinder has been widely utilized in safe driving systems, gesture interfaces of handheld devices, obstacle detection sensors of robots and drones, among others [2]. For example, reversing radar is an essential part of the safe driving system. However, in most automotive applications, the distance measuring system needs a minimum measuring range of more than 5 m [3].

Optical solutions presently dominate in the long-range sensing market. Despite of their relatively high resolution and frame rate, these systems suffer from high power dissipation, typically more than several Watts [4]. Further drawbacks of optical solutions are less immune to ambient illumination and limited performance under strong sunlight conditions [5]. Compared with optical solutions, ultrasonic ranging is not affected by the transparency and color of the target object and can be used directly in sunlight. Besides, because of the relatively low speed of sound, ultrasonic transducer based on ranging technology mitigate the high-speed electronics problems confronted by radio frequency (RF) and optical ranging [6]. Ultrasonic ranging has the characteristic of low power consumption, which makes it an attractive alternative for range applications (e.g., detection distance < 10 m) [7].

For long-range measurements, existing ultrasonic rangefinders typically use bulk piezoelectric materials to generate high-pressure ultrasonic waves. Although range finders of this type usually have good performance and are successfully used in many applications, they suffer from a few significant drawbacks. The piezo-ceramic transducers are expensive and low yield due to its manual fabrication processes [2,8]. Although bulk piezoelectric transducers can generate high output power, a mismatch of acoustic impedances between the devices and transmission medium severely reduces transduction efficiency [9]. An additional matching layer can improve the efficiency, but the method is complicated and only effective in a limited bandwidth [10]. The problems mentioned above limit the applications of bulk piezoelectric ultrasonic transducers in emerging industries, such as consumer electronics and smart home applications.

With the development of micro-electro-mechanical systems (MEMS) technology, micro-machined ultrasonic transducers (MUT) have been extensively studied. MUT has the advantages of small size, low cost, low power consumption, etc., and is considered an alternative to bulk piezoelectric transducers [11]. MEMS-based manufacturing processes have the potential for mass production, which can greatly reduce manufacturing costs and improve sensor uniformity. MUT devices are small in size, high in resonant frequency, and have better impedance matching with the medium due to bending mode operation. MUT devices also have the advantages of being able to form one-dimensional and two-dimensional array structures and facilitating to integrate with supporting electronic devices [12,13,14]. In 2010, Przybyla, R et al. applied piezoelectric micromachined ultrasonic sensors to rangefinders [7]. The PMUT used in the air has excellent performance as a generator and receiver, and it has low manufacturing cost due to the photolithography production process. Also, good SNR can be obtained with limited power consumption when working at the first resonant frequency. It is worthwhile using the low-cost, low-power rangefinder used for most consumer electronics. Therefore, MUT is an encouraging candidate for replacing bulk transducers.

According to the working principle, there are two types of MUT: capacitive micromachined ultrasonic transducer (CMUT) and PMUT. The CMUT is a small capacitor consisting of a thin metalized suspension film (such as silicon nitride) covered in a cavity with a rigid metalized substrate (such as silicon). To achieve a satisfactory sensitivity, the gap is normally designed to be a sub-micrometer [15]. However, it inversely restricts the vibration amplitude of the suspended membrane, thus limiting the output acoustic power. These problems have restricted the application of CMUT in the ultrasonic rangefinder. Compared with CMUT, PMUT does not require an ultrahigh voltage or narrow gap to achieve satisfactory sensitivity [16]. What’s more, PMUT has been well developed with the advantages of a cost-effective manufacturing process, lower power consumption, and good acoustic impedance matching. Air-coupled PMUT is utilized as ultrasonic TOF sensor for range-finding [2,17,18]. Therefore, the PMUT is a promising technology and can be adopted for a wide spectrum of applications.

Various piezoelectric materials, such as thin-film piezoelectric lead zirconate titanate (PZT) [19] and thin-film aluminum nitride (AlN) [20], have been widely applied in PMUT and bulk ultrasonic transducers. The major disadvantages of thin film PZT are its high deposition temperature (e.g., >480 °C) and high poling voltage required in the post process. In comparison, AlN can be deposited at much lower temperatures (≤300 °C) that are compatible with all standard CMOS processes [21,22]. Although PZT shows a relatively higher piezoelectric coefficient, the significant dielectric loss trades off this benefit, and the overall electromechanical coupling coefficient of the PZT device is usually about 50% greater than that of the AlN counterpart [23]. On the other hand, compared to PZT, AlN offers better receiving sensitivity, temperature stability [15]. Moreover, the AlScN significantly boosts PMUT performance in transmitting and receiving ends. AlScN retains many features of AlN (e.g., high receiving sensitivity, ease of deposition/etching, lead-free) possesses but significantly increased piezoelectric properties compared to pure AlN [24,25]. In the past ten years, AlScN PMUT has drawn more and more global research attention and has made breakthroughs in its commercial applications.

In the last decade, ultrasonic rangefinders based on the AlN PMUT have become an increasingly global research interest, and breakthroughs have been achieved in its measurement range. In 2010, Przybyla et al. [7] presented an ultrasonic rangefinder operating range of 30 mm to 450 mm and operated at a 375 Hz maximum sampling rate. In 2012, Przybyla et al. [5] presented a 2D ultrasonic depth sensor based on an array of piezoelectric micromachined ultrasound transducer elements, which measures the range and direction of targets in the air. The maximum measuring range of an ultrasonic rangefinder is 750 mm. In 2014, Przybyla et al. [1] increased the maximum measurement range to 1m. In 2016, Rozenet et al. [26] presented a monolithic MEMS-CMOS rangefinder using PMUT. Using the sensor for pulse-echo range finding, the maximum range of a single PMUT was found to be 980 mm round trip at 411 kHz. In 2021, Chiu et al. [6] presented a high-accuracy CMOS-driven ultrasonic ranging system based on air-coupled AlN-based PMUTs using TOF. The maximum measuring range of the ultrasonic rangefinder is 500 mm, with an average error of 0.63 mm. In 2021, Cai et al. [27] presented an enhanced-differential PMUT based on AlN, which realized ultralong and high-accurate distance measuring. With a resonant frequency of 77.34 kHz, this PMUT has a 40–1000 mm working range base on the TOF measurement principle in the chip-to-chip experiment. The test results show that the maximum measuring error is 4 mm. However, most of the studies mentioned above are focused on the close-range (<5 m) measurements. For other potential applications where conventional air-coupled ultrasonic sensors are used, such as automotive parking assistance, PMUTs have yet to provide sufficient pressure output and receiving sensitivity for a long-range measurement (>5 m).

In this paper, AlScN film-based PMUT is designed for long-range detection. Doping scandium in aluminum nitride is a way to improve the performance of the PMUT [24,25,28,29] and achieve a receive sensitivity of 5.7 mV/Pa. A PMUT array is composed of 14 designed PMUTs that could reach a very high SPL of 120 dB at 10 cm. The range capability of this AlScN PMUT array is evaluated using the designed device working with a commercial range detection system, and the designed device can range more than 6.8 m.

## 2. PMUT Design and Modeling

**Figure 1:**
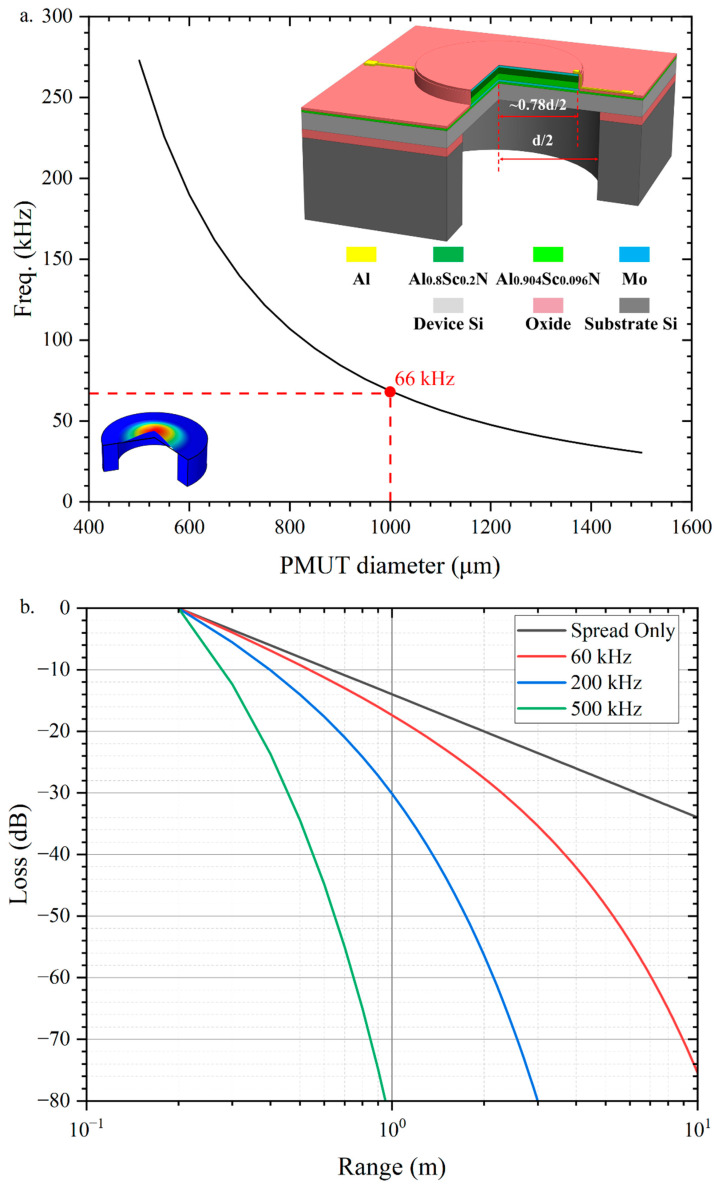
(**a**) The resonance frequency versus the PMUT diameter. The resonant frequency decreases with increasing diameter; (**b**) The path loss of ultrasonic waves of different frequencies transmitted in the air at room temperature of 20 °C with 50% air humidity. The path loss of ultrasonic waves in the air increases with ultrasonic frequency. The ultrasonic wave with 60 kHz resonance frequency, which has the lowest attenuation in air, is selected for long-range detection.

The ultrasonic transducer is composed of a circular monolithic film. It consists of a piezoelectric material sandwiched between two electrodes and a passive bending layer beneath the piezoelectric sandwich. As shown in Figure 1a, the etched tube on the chip exposes both sides of the ultrasonic transducer diaphragm. The electric field generated by the voltage between the top and bottom electrodes causes lateral stress in the piezoelectric layer, which causes the vibration to bend outward and vibrate, creating a pressure wave. Similarly, the incident pressure wave will cause deformation of the diaphragm, resulting in charge on the electrode due to the inverse piezoelectric effect so that the device can be used as both an ultrasonic transmitter and ultrasonic receiver. The diaphragm vibrates in a bending mode to emit ultrasonic waves into the surrounding medium, usually using the basic 00 modes because this mode has the most significant average surface velocity and, therefore, the highest acoustic coupling. The thickness and diameter of the PMUT are selected to achieve the desired resonant frequency, ranging from 40 kHz to 800 kHz for the air-coupled PMUT.

In this work, a circular diaphragm is chosen for the design of the PMUT. The oscillating diaphragm consists of a piezoelectric layer of the thickness ($t_{p}$) and an elastic layer of the thickness ($t_{s}$). The schematic cross-section is shown in Figure 1a. For a plate consisting of a single material [19], the resonant frequency of the PMUT operating in the 00 modes can be calculated by the following equation

(1) $$f_{0}=\lambda_{00}^{2}\frac{t}{4a^{2}}\sqrt{\frac{E^{\prime }}{\rho_{m}}}$$

where $\lambda_{00}^{2}=1.88$ is the characteristic value of vibration mode, the average plate modulus $E^{\prime }=\left(E_{p}^{\prime }t_{p}+E_{s}^{\prime }t_{s}\right)/\left(t_{p}+t_{s}\right)$, $E_{p,s}^{\prime }=E_{p,s}/\left(1-\nu^{2}\right)$, $E_{p,s}$ is Young’s modulus of the piezoelectric layer and Si layer, ν is Poisson’s ratio, $\rho_{m}$ is the average density of PMUT film, a is the radius of PMUT film. Figure 1a shows the bending mode PMUT with the maximum diaphragm displacement at the center of the diaphragm.

The model parameters of the PMUT are similar to those usually used to model capacitive MUT. The electromechanical transformer ratio coefficient is given by [19]

(2) $$\eta =12\pi \gamma^{2}\left(\gamma^{2}-1\right)e_{31,f}z_{p}\;$$

where $e_{31,f}$ is the transverse thin-film piezoelectric coefficient, γ=78% is the ratio of electrode diameter to PMUT diameter, $z_{p}$ is the distance from the neutral axis to the mid-plane of the piezoelectric film. The modal stiffness is $k=192\pi D/a^{2}$, where $D=E^{\prime }t^{3}/12$ is the flexural stiffness. The modal mass is $m=9\pi a^{2}\rho t/5$. The acoustic impedance is given by [1]

(3) $$Z_{pmut}\left(a\right)=\frac{Z_{0}}{A_{m}}\left(1-\frac{2J_{1}\left(4\pi a/\lambda \right)}{4\pi a/\lambda }+j\frac{2K_{1}\left(4\pi a/\lambda \right)}{4\pi a/\lambda }\right)\;$$

where $J_{1}$ and $K_{1}$ are first-order Bessel and Struve functions, λ is the wavelength, and $Z_{0}=413\;Pa.s/m$ is the acoustic impedance of air. The real part of $Z_{pmut}$ acts like a resistance and is equivalent to mechanical damping, while the imaginary part is equivalent to mass and is the origin of the correction factor in (2). The real part of $Z_{pmut}$ reaches a maximum value $Z_{0}/A_{m}$, when the PMUT a>λ/π. $A_{m}=\frac{1}{12}\pi a^{2}$ is the effective area of PMUT. The acoustic damping is equivalent to the mechanical damping in the mechanical domain is $b_{m}=A_{m}Z_{0}$. When PMUT is operating $b_{m}$ is the dominant damping term, so we use this maximum value in the following to simulate the worst case.

The pinned boundary structure is chosen to improve the acoustic performance of the PMUT in vibration amplitude and sound pressure [30]. The pressure output per volt at resonance is improved 3.5 times compared to the PMUT with clamped boundary. To increase the acoustic coupling of the PMUT, an impedance-matched resonant tube etched underneath the PMUT. The SPL is increased by 350% compared to the transducer without the impedance matching tube [31]. In this study, a 4 μm silicon layer ($t_{s}$), and a 1.2 μm piezoelectric layer ($t_{p}$) are selected as the fabrication process for fabrication reasons. For the selected AlScN/Si layer thickness, the variation of resonant frequency with radius is predicted using Equation (1) and the results are shown in Figure 1a.

In rangefinders, the strength of the echo signal depends on the target distance and the acoustic reflectivity. The latter is close because the acoustic impedance of most materials is several orders of magnitude greater than that of air. Since the transducer is described here λ≫a at resonance, the ultrasonic energy radiates isotropically from the front of the transducer, resulting in a linear decay of pressure with distance. In addition, the vibration of air molecules causes an exponential signal attenuation. $\alpha =3.61\times 10^{-6}f_{0}-0.0985$ is the attenuation constant in bels/m and $f_{0}$ is the frequency of the acoustic wave. The vibration loss constant α increases with increasing humidity. According to the ultrasound propagation attenuation model [7], the ultrasound loss (in decibels) with respect to the reference range $R_{0}$ is

(4) $$L\left(R\right)=-20\log\left(R/R_{0}\right)-2\alpha \left(R-R_{0}\right)\;$$

where R is the round-trip distance of the ultrasonic flight, when considering the worst case (air humidity of 100%), the distance attenuation of ultrasonic waves of different frequencies is shown in Figure 1b, among the frequencies generated by the air-coupled PMUT, the ultrasonic waves of 60 kHz have the least compared to the medium frequency ($f_{0}=200\;\mathrm{kHz}$) and the higher frequency ($f_{0}=500\;\mathrm{kHz}$). Therefore, a frequency of about 60 kHz was finally chosen as the operating frequency of the telemeter PMUT. From Figure 1a, the radius of PMUT a=1000 μm. The final PMUT dimensional parameters are summarized in Table 1.

The ultrasonic signal propagates through the air by acoustic gain, target reflection and propagation attenuation. Therefore [1,7], the pressure returned from the target is

(5) $$p_{rx}=\left(\rho_{0}c_{0}V_{in}/b_{m}\right)G_{ac\;total}\;$$

where $\rho_{0}=1.2\;\mathrm{kg}/m^{3}$ is the air density, $c_{0}=343\;m/s$ is the propagation velocity of ultrasonic waves in air, $V_{in}$ is the driving voltage, $G_{ac\;total}=G_{ac}G_{targ}G_{path}$, and $G_{ac}$ is the acoustic gain added by the PMUT. This gain is due to factors such as the back cavity, exposed PCB in air, etc., which exposes the back side of the membrane to air, thus increasing the effective area of the transducer and making it more directional. $G_{targ}$ is the target reflectivity and depends on the size of the geometry. When the target distance is R and the target is perpendicular to the PMUT, for the target surface radius, there is $r_{targ}>\sqrt{\lambda R/\left(2\pi \right)}$ equation λ=5 mm is the wavelength of ultrasonic waves generated by the PMUT. At this time $G_{targ}=1$. $G_{path}$ is the attenuation of ultrasonic waves in the process of air propagation, depending on the ultrasonic frequency, ambient temperature humidity and propagation distance. Assuming that the target reflects the ultrasonic waves ultimately, the ratio of the pressure received at the receiving end to the pressure transmitted is [1,7]

(6) $$G_{path}=\frac{p_{rx}}{p_{tx}}=\frac{\sqrt{A_{m}}}{4\sqrt{\pi }R}10^{-2\alpha R}\;$$

The amplitude of the input signal is [1]

(7) $$v_{rx}=p_{rx}\frac{A_{m}}{\eta \omega_{0}C_{0}R_{m}}\;$$

where $p_{rx}$ is the sound pressure received at the receiver, $\omega_{0}=2\pi f_{0}$ is the angular frequency of the PMUT, $C_{0}$ is the capacitance of the transducer, and $R_{m}=b_{m}/\eta^{2}$ is the motion impedance of the transducer. Since the noise does not depend on the range, the above equation shows that the signal decreases linearly with increasing range over a relatively short range, and then begins to decrease more rapidly as absorption begins to become the dominant loss factor.

In a well-designed ranging system, there are two primary sources of noise: the random thermal (Brownian) motion of the air received by the transducer and the thermal noise from the front-end amplifier. Without considering the circuit noise introduced by the system circuitry, the noise of the ranging system at this point comes primarily from the random thermal motion of the air. The collisions of air molecules with the transducer membrane occur randomly, and the process can be modeled as white noise. This white noise is converted to a noise voltage of ${\bar{V}}^{2}/BW=4k_{b}TR_{m}$ by the piezoelectric effect, which affects the output signal at the PMUT output. Therefore [1,19], the noise voltage at the output of the transducer is

(8) $${\bar{v_{m}}}^{2}=\frac{k_{b}T}{L_{m}\omega_{0}^{2}C_{0}^{2}}\;$$

where $k_{b}$ is the Boltzmann constant, T is the ambient temperature, and $L_{m}=m/\eta^{2}$ is the equivalent inductance of the transducer. From Equations (7) and (8), the SNR at the receiving end of the ranging system, without considering the circuit noise introduced by the system circuit is

(9) $$\mathrm{SNR}=\frac{{\left|v_{rx}\right|}^{2}}{{\bar{v_{m}}}^{2}}=\frac{N_{tx}^{2}N_{rx}m{\left(p_{tx}G_{ac\;total}\frac{1}{\rho c}\right)}^{2}}{k_{b}T}\;$$

where $N_{tx}$ and $N_{rx}$ are the number of transmitters and receivers [1]. The maximum detection distance of the ranging system is calculated using Equation (9), and the maximum detection distance of the system is 6.8–8.4 m when the SNR threshold of the system is 11.5 dB [1].

## 3. Fabrication Process

**Figure 2:**
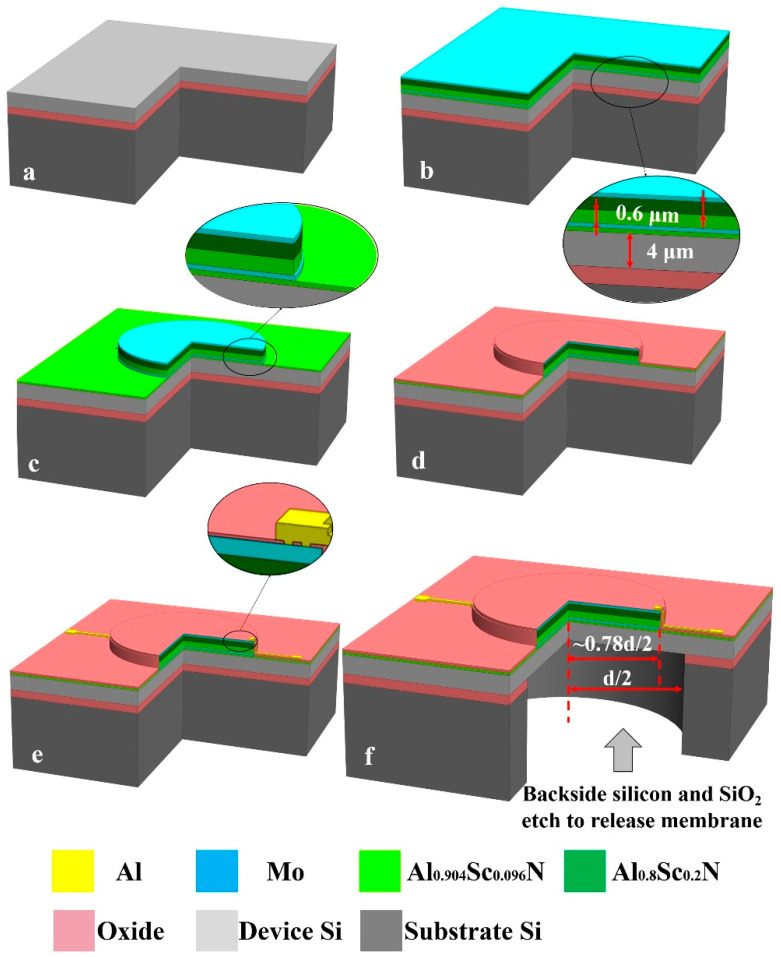
The process flow of pinned boundary hybrid-morph AlScN PMUT: (**a**) Starting with an SOI wafer; (**b**) Sputtering of Al_0.904_Sc_0.096_N/Mo/Al_0.904_Sc_0.096_N/Al_0.8_Sc_0.2_N/Mo stack; (**c**) Pattering of top Mo electrode, Al_0.8_Sc_0.2_N/Al_0.904_Sc_0.096_N piezoelectric layer, and bottom Mo electrode; (**d**) PECVD SiO_2_ deposition and via etching; (**e**) Metallization to complete the interconnection of PMUT; (**f**) Backside substrate silicon and box SiO_2_ layer etch to release membrane.

**Figure 3:**
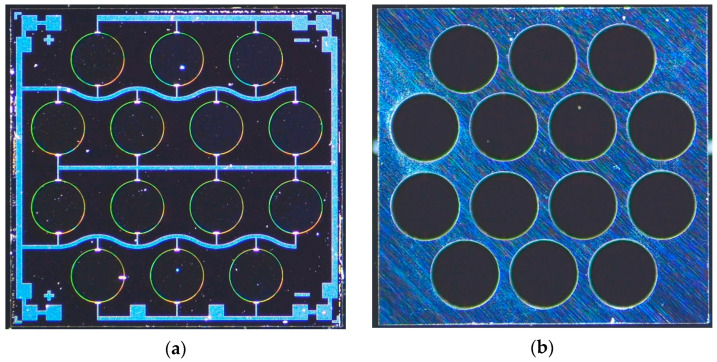
PMUT array optical image: (**a**) Front of PMUT array. The array is composed of 14 designed PMUTs; (**b**) Back of PMUT array.

As shown in Figure 2. The process flow of pinned boundary hybrid-morph AlScN PMUT starts with an silicon on insulator (SOI) wafer (4 μm device silicon layer and 1 μm buried oxide layer). Firstly, sputtering a Al_0.904_Sc_0.096_N/Mo/Al_0.904_Sc_0.096_N/Al_0.8_Sc_0.2_N/Mo stack with thickness-es of 50 nm/200 nm/600 nm/600 nm/100 nm on an SOI wafer. Compared with pure AlN and AlScN film, the hybrid-morph film has a higher piezoelectric coefficient, which addresses the problem of AlScN abnormal grains and a 50 nm thick Al_0.904_Sc_0.096_N film is a seed layer to achieve a good crystalline structure of the subsequent Mo and AlScN layers. The AlScN layers are prepared by reactive magnetron sputtering (Sigma^®^ Deposition System from SPTS, Newport, UK) of an Al-Sc target with a chemical composition of Al_1−x_Sc_x_ (x = 0.096 and 0.2) under a high purity (99.9995%) nitrogen and argon mixture. Secondly, the top molybdenum is patterned using the dry etch process with fluorine-based plasma etch and the maximum displacement is observed with a top electrode radius of 78% of the PMUT radius. Then the AlScN layer is patterned and etched to achieve the pinned boundary condition in the same process as chlorine-based plasma etch. The inductively coupled plasma (ICP, Omega^®^ etch system from SPTS, Newport, UK) etching of AlScN thin films is optimized by adding BCl_3_/Ar pretreatment to the Cl_2_/Ar main etching scheme, improving the selectivity of AlScN to Mo. Similarly, the bottom Mo is patterned. Then 200 nm thick PECVD SiO_2_ is deposited and etched to achieve vias to the top and bottom electrodes. The dry etching in this step must be time-controlled to ensure the Mo layer does not get etched. After via etching, metallization to complete the interconnection of PMUT. The final step is the backside DRIE process to release the membrane and define the radius of PMUT. The final fabricated array is shown in Figure 3. The final array size is 4.5 mm × 4.5 mm and consists of 14 PMUTs with 1000 µm diameter. In this paper, PMUT of pinned structure with piezoelectric layer of 1 µm Al_0.9_Sc_0.1_N passive layer of 5 μm Si are also fabricated as a control for electrical and mechanical tests. The test results are shown in the next section.

## 4. Characterization and Discussion

### 4.1. Electrical Characterization

**Figure 4:**
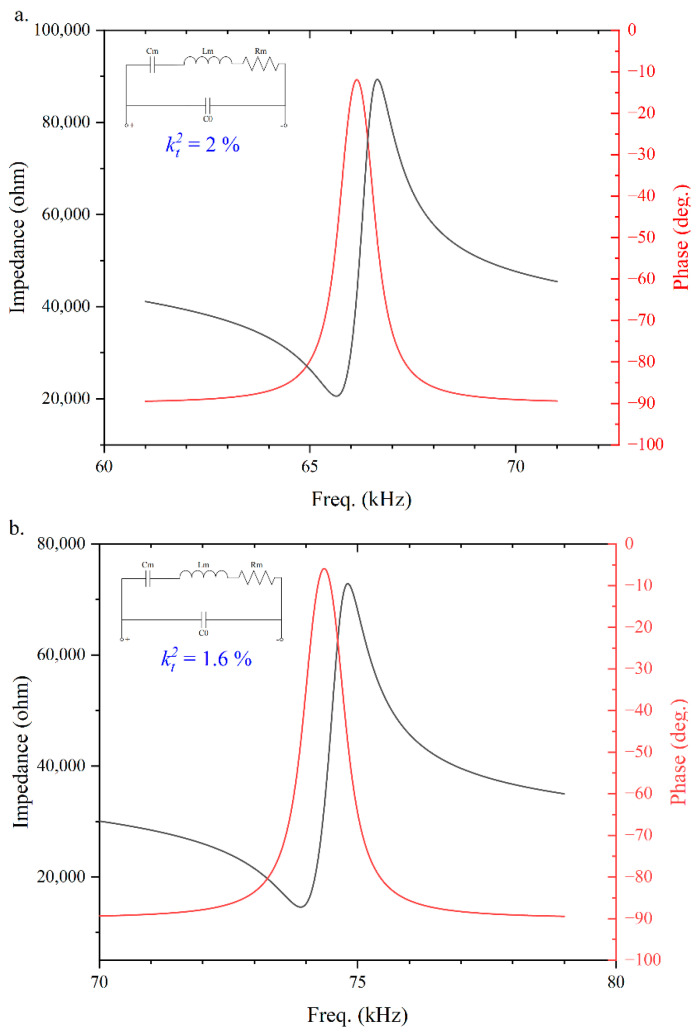
Electrical impedance measurement of (**a**) The designed PMUT with the resonant frequency of $f_{0}=66\;\mathrm{kHz},\;k_{t}^{2}=2\%$; (**b**) The control PMUT with the resonant frequency of $f_{0}=74\;\mathrm{kHz},\;k_{t}^{2}=1.6\%$.

The electrical parameters of the PMUT are measured by an impedance analyzer (KEYSIGHT, Beijing, China). Figure 4 shows the electrical characteristics of a single PMUT element. The electromechanical coupling factor ($k_{t}^{2}$) can be estimated by approximating the following equation

(10) $$k_{t}^{2}=\frac{C_{m}}{C_{m}+C_{0}}$$

where $C_{m}=1.14\;\mathrm{pF}$ is the equivalent capacitance of the PMUT, and $C_{0}=56.4\;\mathrm{pF}$ is the parasitic capacitance of the PMUT. The calculated $k_{t}^{2}$ is 2%. The different thickness of the elastic layer Si leads to a deviation in the frequency of the PMUT with the same structure and radius. High doping of scandium result in larger $k_{t}^{2}$, makes the energy conversion more efficient, thus cause produce larger output sound pressure.

### 4.2. Mechanical Characterization

**Figure 5:**
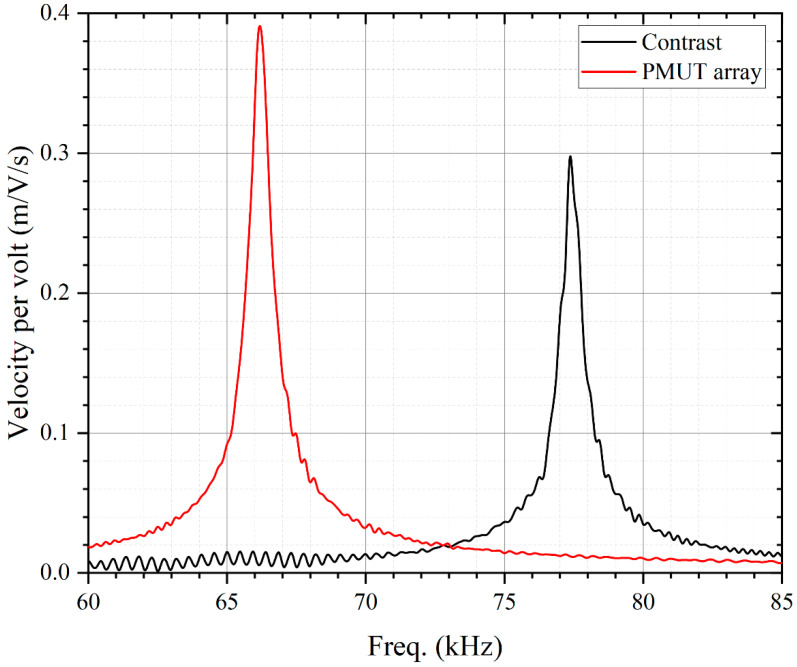
PMUT mechanical characterization: Frequency domain velocity per volt characterization of the PMUT with LDV. The result shows that the velocity of the designed PMUT (**red**) is 30% higher than that of the contrast PMUT (**black**), indicating that the designed PMUT has higher transmit sensitivity and can produce higher output sound pressure.

The PMUT mechanical characteristics are measured by a single-point laser Doppler vibrometer (LDV). Figure 5 shows the velocity-frequency response of the PMUT with a radius of 500 μm. The red curve in Figure 5 shows that the designed PMUT has a resonant frequency of 66 kHz and a velocity of 0.39 m/V/s per volt, which is proportional to the sound pressure. In this paper, the vibration velocity per volt of the designed PMUT is 30% higher than that of the contrast PMUT (black). The designed PMUT can produce higher sound pressure, which results in a longer measurement range.

### 4.3. Acoustic Characterization

**Figure 6:**
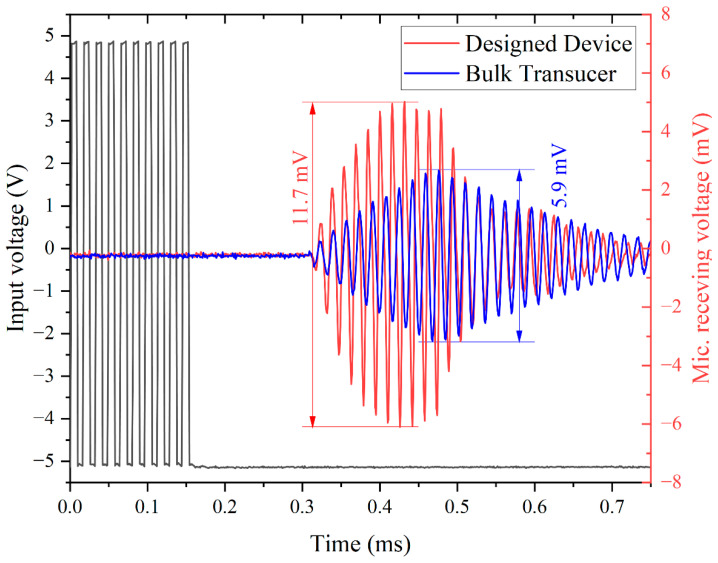
Acoustic time-domain characterization: acoustic measurements use a designed PMUT array and a calibrated reference microphone. The PMUT array is excited by 10-cycle square continue wave with driving voltage of 10 $V_{pp}$. The sound pressure of 20.35 Pa (120 dB SPL, red) is measured at 10 cm, which is 9.7 Pa (114dB SPL, blue) for the bulk transducer.

Figure 6 shows the results of a one-transmission experiment performed by a PMUT array and a calibrated reference microphone (B&K model 4138, Denmark). The PMUT array is excited by 10 cycles, 10 $V_{pp}$ pulsed continuous wave drive at 66 kHz, the pressure transmitted from the PMUT array measured by the microphone generating 20.35 Pa (120 dB SPL) at 10 cm. The experimental results are consistent with the theoretical calculations in Equations (4) and (5). The sound pressure of 9.7 Pa (114 dB SPL) at 10 cm is obtained in the same situation using the bulk transducer MA58MF (Murata Manufacturing). The bulk transducer MA58MF is a closed-top transducer and therefore requires sufficient drive voltage to produce a large sound pressure.

**Figure 7:**
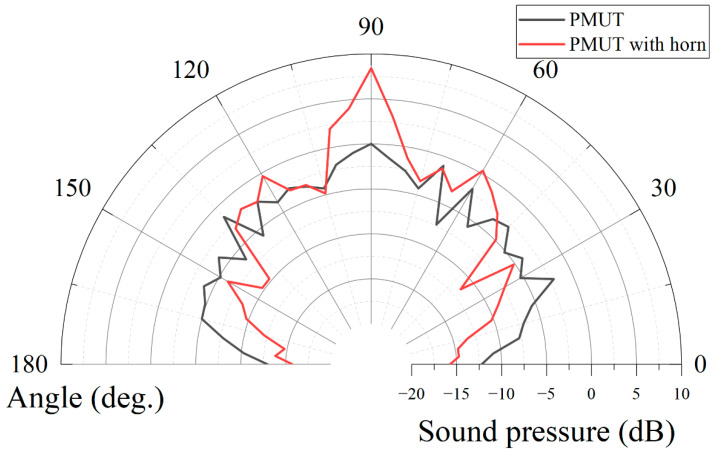
Directivity characterization of PMUT array: the directivity without horn (**black**), half-power-beam-width θ_−3dB_ is 100°. The directivity of PMUT with horn (**red**), half-power-beam-width θ_−3dB_ is 20°. Adding the horn can focus the energy to increase the output sound pressure and reduce the sound field angle to avoid unnecessary objects during the long-distance measurement.

The results of the directivity of the device are shown in Figure 7. The half-power-beam-width θ_−3dB_ of the PMUT array is about 100°. However, this results in the dispersion of acoustic energy and reduces the sound pressure of the transducer in a single direction. In this paper, a horn is added to the PMUT array to concentrate the acoustic energy and increase the sound pressure of the transducer in the direct front. As shown in the black line of Figure 7, the presence of the horn reduces the half-power-beam-width θ_−3dB_ of the PMUT array by 80%, concentrates the sound energy, and increases the sound pressure of the transducer in the front. The smaller sound field angle is more favorable for long-range ranging experiments because the objects that interfere during the experiment can be avoided.

**Figure 8:**
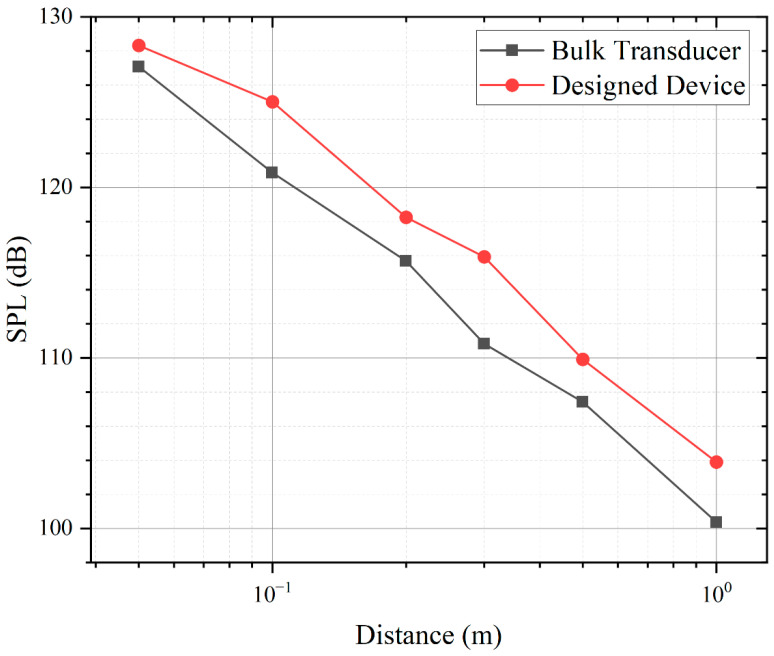
PMUT SPL pressure output versus measurement distance. The designed device (**red**) is driven by a 10 $V_{pp}$ continuous square wave transmitting 125.5 dB SPL at 66 kHz at 10 cm 5 dB higher than the bulk transducer (**black**).

The SPL measurements of the PMUT array and the bulk transducer are shown in Figure 8. The transducer is driven by a continuous square wave of 10 $V_{pp}$ at resonant frequency conditions, using a reference microphone at a distance of 5 cm to 1 m. The addition of the horn allows the PMUT array to produce a larger sound pressure than the bulk transducer, which allows the PMUT array designed in this paper to achieve a longer measurement range. Table 2 shows the comparison between the PMUT array and the bulk transducer. The PMUT array can produce higher sound pressure with 80% smaller area, which indicates the better performance of the PMUT.

## 5. Rangefinding Experiments

**Figure 9:**
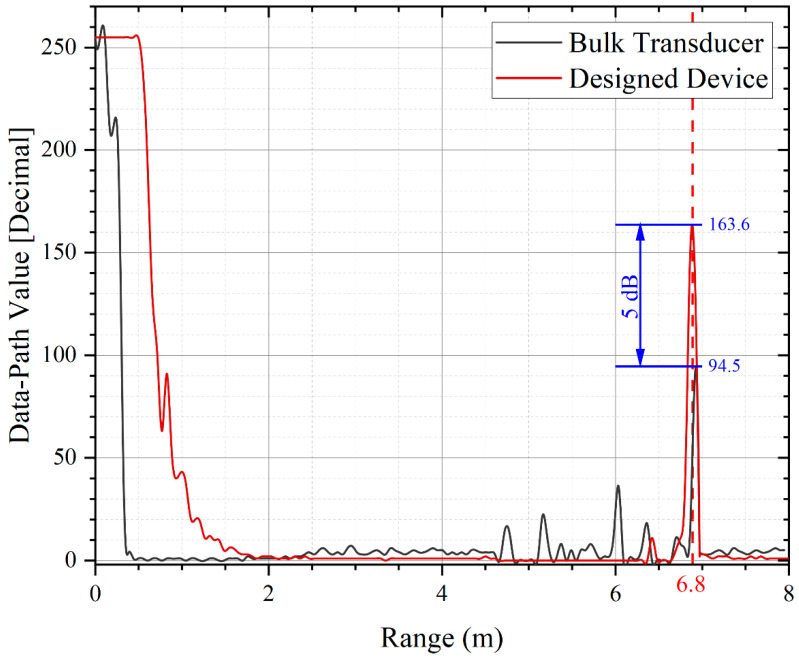
Long-range detection results. When the detection distance is 6.8 m, the signal output of the designed device (**red**) is 5 dB larger than that of the bulk transducer (**black**).

**Figure 10:**
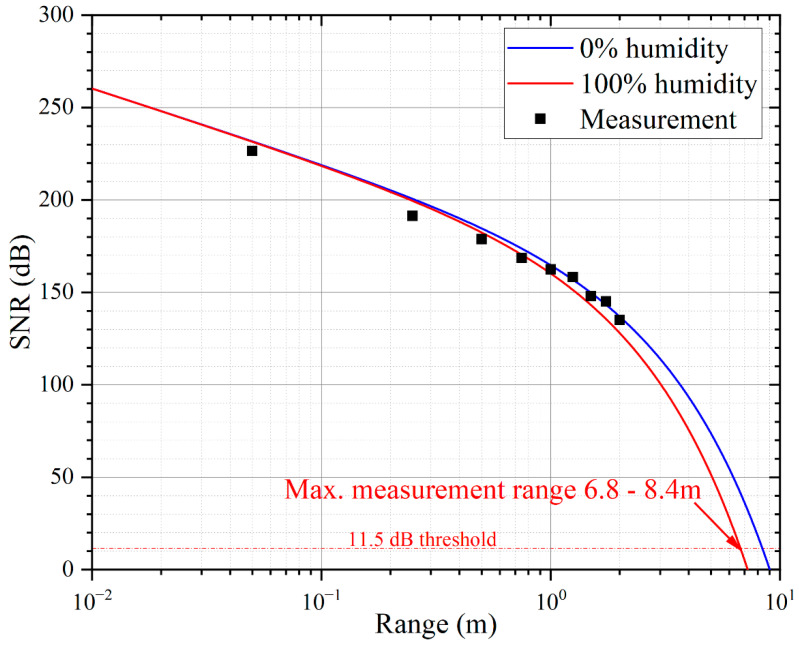
SNR of long-range detection system versus measurement distance. The SNR theoretical model shows the maximum measurement range of the designed device to be 8.4 m at 0% air humidity (good environment, **black**) and 6.8 m at 100% air humidity (bad environment, **red**).

The telemetry experiments are conducted with a single PMUT array, which can avoid the loss caused by frequency mismatch. The PMUT array is placed on a PCB with a 5 mm thick baffle plate with an area of 1 × 1 m^2^ at the distance of 1 m to 7 m. The PMUT array works with a commercially available distance detection system (made by TI PGA460), transmitting ultrasonic waves under 30 pulses at 66 kHz. The ultrasonic waves are reflected by the target baffle and the echoes are received by the PMUT array. The electrical signal is processed by the range detection system and the result displayed on user interface. Figure 9 shows an example of the results of long-range detection. The designed device (red) detects a signal amplitude of 163.6 at 6.8 m, which is 5 dB higher than the bulk transducer (black), and the distance detection results are consistent with the results of the acoustic characterization study in the previous chapter. This experiment achieves a long-range detection of 6.8 m, indicating a detectable distance of 1.36 times that of other devices reported before [32]. Figure 10 summarizes the theoretical calculation and measurement results of the SNR. The theoretical maximum measuring range of the device is 8.4 m and 6.8 m when the air humidity is 0% (good environment, blue) and 100% (bad environment, red). Table 3 compares the performance of the designed device with the different PMUT [19,32]. The designed device in this study achieves the highest receive sensitivity with AlScN and the longest measurement distance.

## 6. Conclusions

This study has achieved long-range detection beyond 5 m. The designed PMUT is made of Al_0.8_Sc_0.2_N as the piezoelectric layer with a resonant frequency of 66 kHz to mitigate ultrasonic attenuation in air. The PMUT array for long-range detection is composed of 14 designed PMUT elements which can achieve a high receive sensitivity of 5.7 mV/Pa. Acoustic characterization shows the PMUT array generates a very high SPL of 120 dB at 10 cm, revealing its potential for achieving a better range-finding performance than that of conventional bulk transducer under the same system condition. The long-range detection experiment is conducted using our PMUT array with a decent sensing performance recorded even at distance of 6.8 m and outperforms the bulk transducer counterpart by 5 dB in term of system-level SNR. By and large, our experiments validate the proposed AlScN MEMS PMUT device as a promising candidate for most long-range ultrasonic proximity sensing application.

## Figures and Tables

**Table 1:** PMUT material characteristics.

Materials	Wide [μm]	Height [μm]	E [GPa]	𝛎	𝛒 [kg/m^3^]	*e*_31,*f*_ [C/m^2^]
Mo	780	0.2	320	0.38	10,220	-
Al_0.8_Sc_0.2_N	780	1.2	230	0.23	3520	−1.6
Si	-	4	168	0.28	2330	-
Tube	1000	600	-	-	-	-

**Table 2:** Comparison of our device and bulk transducer.

Devices	Area [mm^2^]	Height [mm]	Freq. [kHz]	SPL [dB]
This work	19.35	0.6	66	125.5
Bulk Transducer	154	7	58.5	120.5

**Table 3:** Comparison of different PMUT reported before.

Devices	Device Type	Freq [kHz]	Piezoelectric Material	SPL ^1^ [dB]	RX [mV/Pa]	Max Range [m]
This work	array	66	Al_0.8_Sc_0.2_N	125.5	5.7	6.8
[1]	array	217	AlN	58	1.2	1
[2]	single	180	PZT	95	2.1	1
[19]	array	48	PZT	117	1.9	2.4
[26]	array	142	AlN	74	not specified	0.98
[32]	single	82	AlN	not specified	not specified	5

^1^ SPL generated by the device as the transmitter at 10 cm.

## Data Availability

Data are available from the authors on request.
